# Directional nanotopographic gradients: a high-throughput screening platform for cell contact guidance

**DOI:** 10.1038/srep16240

**Published:** 2015-11-17

**Authors:** Qihui Zhou, Philipp T. Kühn, Thirsa Huisman, Elsje Nieboer, Charlotte van Zwol, Theo G. van Kooten, Patrick van Rijn

**Affiliations:** 1 Biomedical Engineering Department-FB40, University of Groningen, University Medical Center Groningen, A. Deusinglaan 1, 9713 AV, Groningen, The Netherlands.; 2 W.J. Kolff Institute for Biomedical Engineering and Materials Science-FB41, University of Groningen, University Medical Center Groningen, Antonius Deusinglaan 1, 9713AV Groningen, Netherlands; 3 Zernike Institute for Advanced Materials, University of Groningen, Nijenborgh 4, 9747 AG, Groningen, Netherlands

## Abstract

A novel approach was developed using PDMS-substrates with surface-aligned nanotopography gradients, varying unidirectional in amplitude and wavelength, for studying cell behavior with regard to adhesion and alignment. The gradients target more surface feature parameters simultaneously and provide more information with fewer experiments and are therefore vastly superior with respect to individual topography substrates. Cellular adhesion experiments on non-gradient aligned nanowrinkled surfaces displayed a linear relationship of osteoblast cell adhesion with respect to topography aspect ratio. Additionally, an aspect ratio of 0.25 was found to be most efficient for cell alignment. Modification of the surface preparation method allowed us to develop an approach for creating surface nanotopography gradients which innovatively provided a superior data collection with fewer experiments showing that 1) low amplitude with small wavenumber is best for osteoblast cell adhesion 2) indeed higher aspect ratios are favorable for alignment however only with features between 80–180 nm in amplitude and 450–750 nm in wavelength with a clear transition between adhesion and alignment efficiency and 3) disproved a linear relationship of cell adhesion towards aspect ratio as was found for single feature substrate analysis.

Cells sense and respond to micro/nanotopographical signals through a process known as contact guidance^1^,^2^,^3^. This topography-sensing process regulates various cellular functions such as cellular signaling, adhesion, morphology, orientation, migration, proliferation and differentiation^4^,^5^. Controlling cell-topography interactions is pivotal for biomaterials design in tissue repair or tissue regeneration and medical implants^6^. The relationship between the topography and cell response depends on the pattern and dimensions, among other factors, which are critical issues to be clarified. Initially, many of these investigations used discrete substrates with different and randomly selected degrees of topography^7^,^8^,^9^, which provided only limited information and lacked in identifying critical parameters that regulate topography-driven cell adhesion and morphology, nor any mimicking tissue microenvironments with heterogeneous (gradient) topography (e.g. cortical-cancellous bone^10^ and osteochondral tissue^11^). Clarifying the correlation between topography and sensing processes requires rigorous sampling and cell experiments, which are time-consuming, costly and suffer from experimental variations in culture conditions.

Surface topographic gradients are valuable platforms for the high-throughput screening of colloidal particle alignment^12^ and optical nanotechnological applications^13^ but also for cell-surface interactions. Using gradients saves time and resources, maximum data output with minimal number of experiments is obtained and variations due to systematic errors minimized. Faia-Torres *et al.* developed a roughness gradient with a biodegradable polymer as a platform to study the roughness dependent osteogenesis induction in human bone marrow-derived mesenchymal stem cells (hBM-MSCs) and found that roughness (average roughness: R_a_ ~ 2.1–3.1 μm; mean distance between features: R_sm_ ~ 71.1–48.1 μm) accelerated the osteogenic commitment of hBM-MSCs and strongly strengthened the osteogenic differentiation of these cells^14^. Kim *et al.* reported a systematic study on how micron-sized surface structure density variations in topography spatially guide the organization and migration of fibroblasts. For this study, gradient surfaces of micron-sized grooves were used with a range of pattern dimensions. The approach was easily incorporated into a single culture platform using ultraviolet-assisted capillary force lithography which could not be accomplished easily with other methods^15^,^16^. Voelcker *et al.* found that SK-N-SH human neuroblastoma cells were sensitive to nanoscale surface topography with feature sizes of <20 nm^17^ and osteogenesis of rat mesenchymal stem cells (rMSCs) was enhanced by porous topography with a ridge roughness lower than 10 nm which was established by screening silicon porosity gradients^18^. Evidently, these previous studies collectively demonstrated that surface topographic gradients are a powerful format that enables cell behavioral studies in a complex but precisely defined microenvironment. However, most studies focused on the microscale alignment gradients or non-directional nanotopographic surfaces and did not contrast cell behavior on topographic gradients in the range between 0–1000 nm including a surface directionality.

Investigating nanoscale structured substrates efficiently is highly important for biointerface investigations as the golden principle for biointerface design is to correlate the features to the natural extracellular matrix (ECM)^19^. Extracellular matrices of e.g. blood vessel, tendon, nerve, have regular and anisotropic architectures consisting of well-aligned micro/nanoscaled fibrous patterns. Individual collagen fibers in the ECM tend to have feature diameters around 150 nm and actual collagen fiber bundles that native cells populate have feature diameters of ~550–900 nm^20^,^21^ depending on the tissue type. Therefore, identifying cell behavior on the nanoscale related to biological structures is of great importance. We developed arrays of parallel nanowrinkles on PDMS (polydimethylsiloxane) substrates which mimic the ECM size features of anisotropic tissues and thereby capturing many of the details that are not revealed in micron-sized grooves^22^ or wrinkle^23^.

Previous studies also focused on the effects of the substrate nanotopography on cell functions^22^, however, without specific alignment and not in a gradient fashion as is shown here. We observed that osteoblasts which are aligned onto surface features mimicking ECM dimensions, do not adhere to these surfaces as strongly as on non-aligned ones, as assessed by focal adhesions. Additionally, developing novel approaches that can efficiently identify cell responses towards surface features. Such endeavors are highly relevant for both the biomedical field and fundamental cell biology as recent evidence showed that aligned surface features also influence bone anisotropy^24^,^25^. The uni-directional gradient platform approach developed here allowed visualization of a multitude of situations within a single experiment enabling to extract a better founded conclusion than using single substrate feature studies. The approach was used to study the effect of nanotopography on osteoblast responses towards focal adhesion, cell adhesion, and morphology changes (e.g. orientation) which proved vastly superior with respect to the use of non-gradient substrates. The morphological and directional alteration of cells is essential for appropriate structural construction of tissues and organs. In particular, osteoblast alignment is crucial for the realization of anisotropic bone tissue microstructure and integration of surface features which stimulate this behavior would enhance implant integration. Although, the data-set presented focusses on osteoblasts, the generated platform is not limited to this cell type and future investigations will include various cells endogenous to other tissues which are known to have intrinsic anisotropy.

## Results and Discussion

### PDMS gradient wrinkle formation and characterization

**Figure 1:**
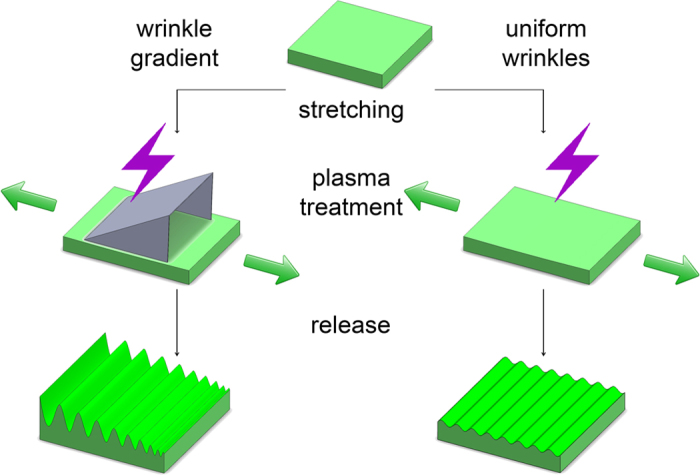
Schematic diagrams of formation of uniform wrinkle and gradient thereof by air plasma oxidation.

In order to omit deviations in experiments due to systematic errors along with minimizing the number of time consuming and costly experiments, a novel approach was developed using unidirectional nanotopography gradients. This approach provides a multitude of experimental outcomes with a limited number of experiments and thereby offering a more detailed overview of important nanotopography contributions towards cell-surface interactions. The aligned PDMS surface structure is formed by applying a unidirectional strain (30% elongation) during surface oxidation using air plasma (shown in Fig. 1) for different times (50 s, 150 s, 250 s and 450 s) to vary the degree of oxidation. After release of strain, wrinkled surface topographies emerge. Wrinkle amplitude and wavelength increase with increasing plasma oxidation time which is caused by increasing the thickness of the silica-like layer, thereby increasing the stiffness^26^. Surface wrinkle gradients were fabricated using a shielded plasma oxidation by applying a right angled triangular prism mask which is open on the face side on top of the PDMS substrate (Fig. S1). It has to be noted that all samples were post-treated with air plasma after wrinkle formation for 10 min to fully oxidize the surface and thereby excluding any chemical or stiffness variations.

Compared to other methods, such as photolithography^4^, colloidal lithography^27^, polymer demixing^28^, and nanoimprinting^29^,^30^ that were used to create micro/nanotopographic features on substrates, the method offers a rapid and cost-effective procedure of producing topographical gradient nanopatterns on transparent, biocompatible polymeric materials with high pattern fidelity and physical integrity. Previously, Hiltl and coworkers showed such an approach for colloidal particle alignment screening allowing for efficient identification of particle surface interactions. However, there a multitude of simultaneously changing features were used which is not appropriate for cell surface adhesion studies. By closing the sides of the mask, a unidirectional variation is induced rather than a dual directional change as was previously performed^12^.

**Figure 2:**
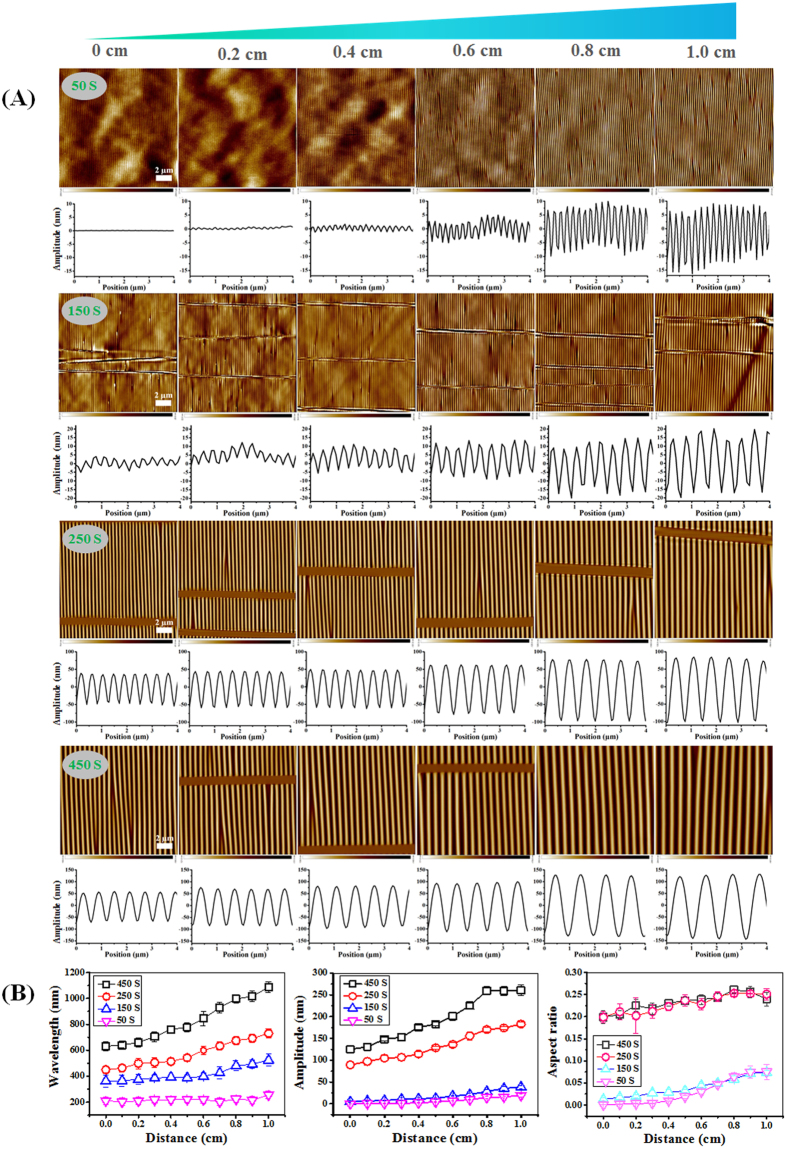
(**A**) AFM images and amplitude curves of the structured PDMS surfaces, via strain-oxidation-release procedure with different oxidation times (0–450 s) and the mask (30°) using air plasma along the 1.0 cm wrinkle gradients. (**B**) Dependence of the wavelength, amplitude and aspect ratio of created wrinkles on the distance with a fixed initial strain (30% substrate elongation and 50–450 s plasma oxidation). Data are reported as mean ± standard deviation (SD) (n = 30 wrinkles). Scale bars are 2 μm and apply to all images.

The surface features of PDMS wrinkle gradients were visualized using atomic force microscopy (AFM), with measurements acquired between 0 and 1.0 cm with 0.2 cm intervals (Fig. 2A). The wrinkle features increased from the least exposed side to the most exposed side (at mask opening) and oxidation times were varied between 50 s to 450 s allowing for even more control over variations in nanotopography gradients. Partial shielding of the substrate using the mask during plasma oxidation for stretched PDMS is crucial to obtain uni-directional gradients with amplitudes ranging from 0.1 nm (Fig. S2) to 260 nm and wavelengths between 200 and 1087 nm as shown in Fig. 2B. Both amplitude and wavelength display a continuous gradual change, the aspect ratio ranges from 0.08 to 0.28. A similar range was observed for the uniform wrinkled substrates, however the major advantage for the gradients is that more amplitude-wavelength combinations are associated to similar aspect ratios and that values for either amplitude or wavelength overlap and therefore contributions from amplitude or wavelength are better assessed. The wavelength, amplitude and aspect ratio for the wrinkle gradients increased with increasing the plasma oxidation time (Fig. 2B). Additionally, the nanopatterning size range obtained in this study encompasses the size of individual collagen fibers present in the native extracellular matrix as well as collagen fiber bundles^20^,^21^. The newly developed gradient surface thus provides a biomimetic platform for studying the effects of surface features in the same size range as for the ECM on the adhesion, morphology and alignment of osteoblasts. Additionally, it has to be noted that because the gradient is developed over 1 cm, the individual changes below a single cell from one end to the other is marginal.

### Screening osteoblast behavior on nanotopography gradient surfaces

**Figure 3:**
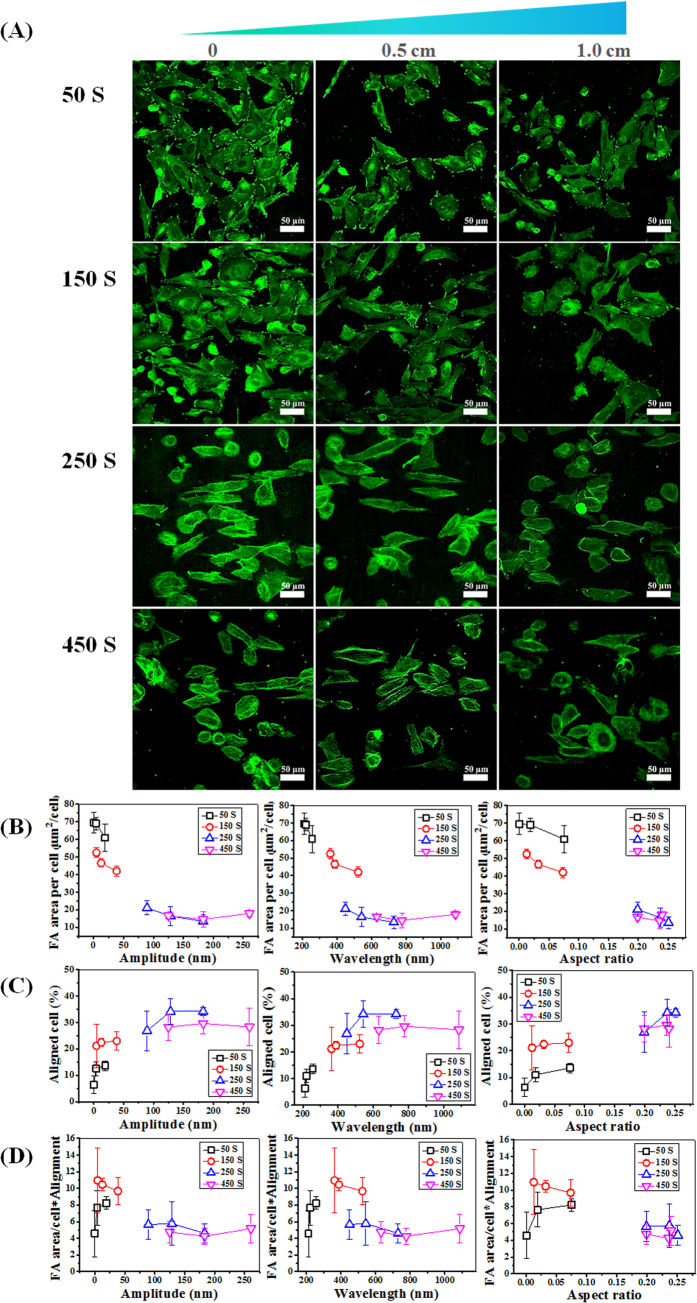
The osteoblast response to the local size of topographic pattern arrays. (**A**) Fluorescent staining of osteoblast vinculin (green) for different wrinkle gradients prepared by varied plasma oxidation times. All wrinkle direction is horizontal. (**B,C**) Dependence of focal adhesion area per cell and aligned cell on amplitude, wavelength and aspect ratio, respectively. (**D**) Dependence of the values of FA/cell * alignment on amplitude, wavelength and aspect ratio. Data reported as mean ± standard deviation (SD) (n = 100 ~ 150 cells).

To study cellular response towards wrinkles, osteoblasts were seeded on the surfaces and allowed to attach and spread for 2 days (Fig. 3A). The formation of focal adhesion contacts was analyzed using immuno-labeling for fluorescent staining and confocal laser scanning microscopy (CLSM). Four different gradients we used to study the effect of surface topography on cell attachment and morphology i.e. cell alignment. Cells were considered aligned if the angle between the long axis and the wrinkle was less than 10° 31. Vinculin staining of osteoblasts on the gradient surfaces displayed that focal adhesion, cell density and morphology are significantly affected depending on the position on the gradient surface (Fig. 3A). For a better understanding of cell behavior on different substrates, focal adhesion area per cell (FA area/cell; μm^2^/cell) was determined by a quantitative analysis of the positively stained focal adhesions. Likewise, focal adhesion orientation and cell alignment were determined measuring the angle of focal adhesion and cell relative to the direction of the wrinkles using ImageJ software (Fig. 3B,C). On the ^0−1.0^ ^cm^50S_grad_ and ^0−1.0cm^150 S_grad_ substrates (position on gradient varies between 0 and 1.0 cm; feature development depends on plasma oxidation times (S)) osteoblasts displayed vinculin-rich FAs, although their number and area were lower and smaller on ^0−1.0^ ^cm^150S_grad_. It was difficult to visualize FAs on the ^0−1.0cm^250S_grad_ and ^0−1.0cm^450S_grad_ surfaces and could only be identified via the quantification procedure as described in the method section (Fig. S3). Additionally, it was found that cell adhesion was sensitive to the topographic gradients, resulting in a cell density gradient along the substrate with ^0−1.0cm^50S_grad_ and ^0−1.0cm^150 S_grad_ probably due to variations in cell adhesion capabilities and cell migration^32^. Cells are able to sense a gradient and can make a migration directional response (haptotaxis), although this was not included in the investigations here. Anchorage-dependent cells on such a haptotactic gradient may move towards the more adhesive region where they accumulate. For ^0−1.0cm^250S_grad_ and ^0−1.0cm^450S_grad_ oxidation surfaces, cells grown on nanopatterned substrates exhibited the typical cobblestone morphology for some cells which was more pronounced on the wrinkles with larger features. Additionally, as the wrinkle size on the gradients increased, cell alignment improved. Among the nano-structured surfaces, ^0−1.0cm^250S_grad_ and ^0−1.0cm^450S_grad_ oxidation surfaces had a better osteoblast alignment, between 30–35% of the cells being aligned, as compared to ^0−1.0cm^50S_grad_ and ^0−1.0cm^150S_grad_ surfaces displaying a maximum alignment of 15 and 25% of the cell population, respectively. From the study using the gradients, it became clear that the most efficient alignment (>25%) occurs with an aspect ratio of about 0.24 but only with wrinkle features between 80–180 nm amplitude and 450–750 nm in wavelength. Similar aspect ratios originating from different amplitude/wavelength combinations displayed altered alignment efficiencies. Although, higher aspect ratios induced better alignment than the lower aspect ratios, it shows that specific feature combinations dictate the cell behavior even when the relative feature ratio (aspect ratio) is similar.

In previous studies, the morphology of cells on surfaces has been investigated^5^,^33^ demonstrating that a close correlation exists between the cell morphology and cell function. It is generally accepted, with few exceptions, that a small, round cell shape is typically indicative of a cell entering apoptosis^5^,^34^, whereas a well-spread and aligned cell shape is most often quantified as being in a viable state. It is possible to quantify cell state by calculating focal adhesion area per cell and measuring the aligned cell by means of imaging software packages^35^. From such a quantitative analysis (Fig. 3B), our study revealed focal adhesion area per cell decreased from 69.6 to 14.5 μm^2^/cell with increasing amplitude, wavelength and aspect ratio. However, cell alignment first increased from 6% which can be considered as random, to 34% and then decreased to 28% with increasing amplitude, wavelength or aspect ratio (Fig. 3C). The wrinkles with 130–180 nm amplitude and 550–730 nm wavelength is the most favorable topography for improving the osteoblast alignment.

The different gradients have overlapping features where similar wavelengths are obtained but with different amplitudes or vice versa. By comparing the overlapping regions one can only conclude that both the amplitude and wavelength, as reflected in the aspect ratio, have a strong influence on the cell adhesion and cell alignment. The adhesion and alignment display opposite behavior in the sense that when adhesion is optimal, alignment (cell morphology) is negatively affected. Both will influence the cellular functions and therefore a combined parametric interpretation allows to identify the most optimum situation e.g. high adhesion strength (more focal adhesion)^36^ combined with high alignment efficiency (morphological control). For adhesion/alignment parameter identification, the values for FA and cell alignment were combined (Fig. 3D, (FA/cell)*alignment%) and an optimum situation was identified where a still high cell adhesion strength was achieved with a relatively high cell alignment %. The optimum was achieved with substrate topography features of amplitude between 15–45 nm and wavelengths between 400–520 nm.

The use of a topography gradient allows for enhanced evaluation of cell behavior to screen the optimal conditions, as compared to uniformly patterned surfaces. For uniform wrinkles, one sample provides only one result of one situation while for gradient surfaces, one sample displays a multitude of situations. As a control, uniform wrinkled surfaces have been used to verify the gradient approach and to confirm the strength of such an approach.

### PDMS uniform wrinkle formation and characterization

**Figure 4:**
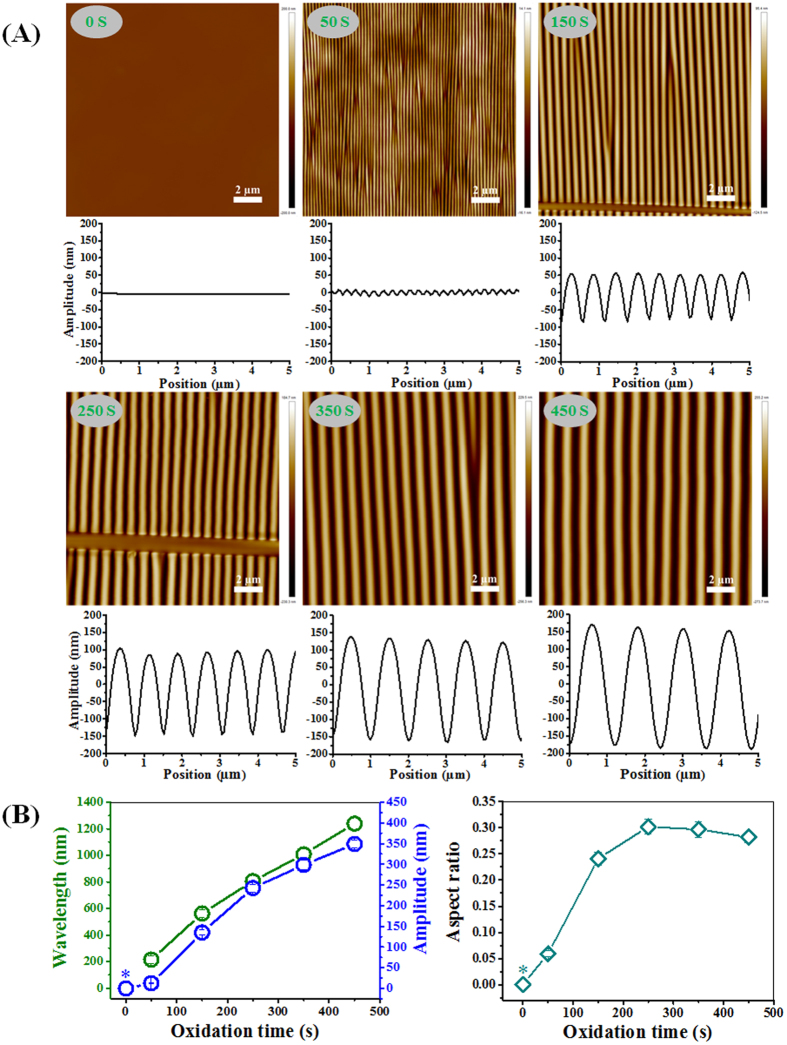
(**A**) AFM images and amplitude curves of the structured PDMS surfaces, via strain-oxidation-release procedure with different oxidation times (0–450 s) using air plasma. (**B**) Dependence of the wavelength, amplitude and aspect ratio of created wrinkles on the plasma oxidation time with a fixed initial strain (30% substrate elongation). * indicates approximate value. Data are reported as mean ± standard deviation (SD) (n = 30 wrinkles). Scale bars are 2 μm.

The uniform aligned PDMS surface structures have been prepared similar as for the gradients but without shielding (shown in Fig. 1) for various oxidation times (50 s, 150 s, 250 s, 350 s and 450 s). Atomic Force Microscopy (AFM) was used to characterize the wrinkle geometries (Fig. 4). Also the uniform wrinkle substrates were post-treated with air plasma after wrinkle formation for 10 min to fully oxidize the surface and thereby excluding any chemical or stiffness variations. Fully oxidized PDMS without applying unidirectional strain was used as a control substrate.

Figure 4B displays the wrinkle wavelength, amplitude and aspect ratio versus the plasma oxidation time. The wrinkles have wavelengths in the range of 215 nm to 1239 nm, amplitudes in the range of 13 nm to 349 nm and aspect ratios in the range of 0.06 to 0.30. The wavelength and amplitude of the wrinkles increase in a linear-like fashion with increasing oxidation time. The aspect ratio initially increases from 0 to 0.25 with longer oxidation time (0–250 s) and then becomes relatively independent of the oxidation time (250–850 s; data for 550–850 s shown in Supplementary Fig. S4) and reaches a plateau value of about 0.30. This is in good accordance with previous observations^37^,^38^.

### Osteoblast response toward uniform wrinkled surfaces

**Figure 5:**
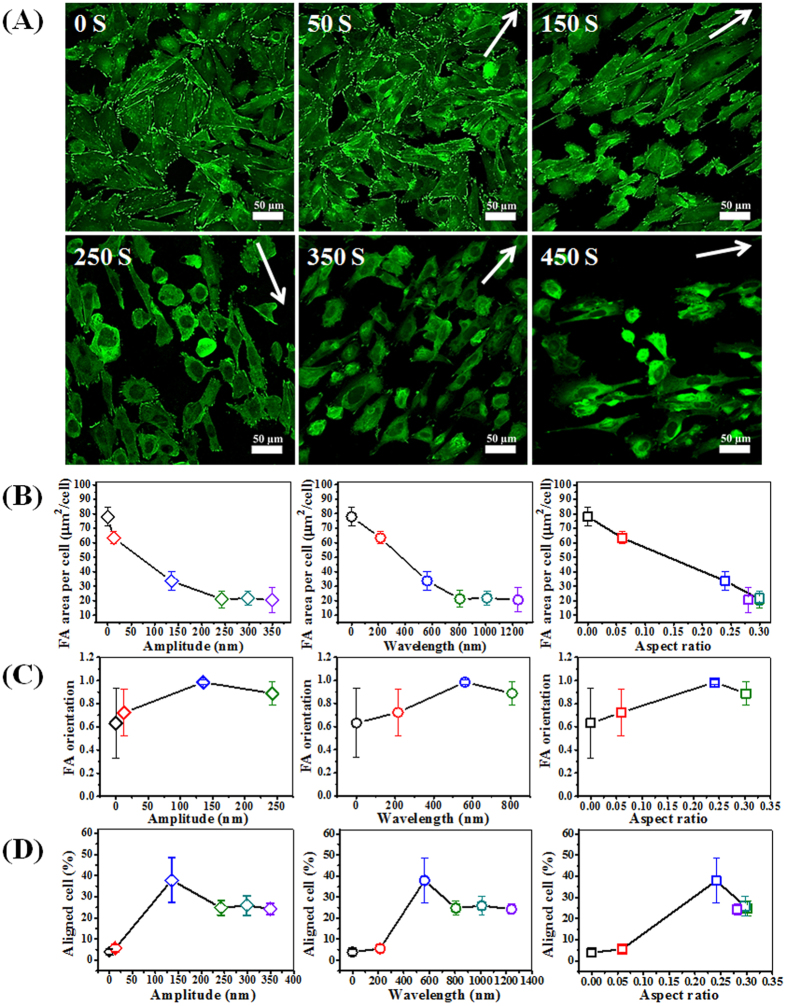
(**A**) Surface adhesion (focal adhesion, visualized using vinculin staining; green) analysis of osteoblasts by confocal fluorescence microscopy on different wrinkle features as shown in Fig. 3. (**B**–**D**) Dependence of focal adhesion area per cell (B), focal adhesion orientation (**C**) and cell alignment (**D**) on amplitude, wavelength and aspect ratio, respectively. Data are reported as mean ± standard deviation (SD) (n = 100 ~ 150 cells). For Fig. 4C, focal adhesion orientation for 350 S and 450 S could not be identified properly and are therefore data points were omitted. Color coding corresponds to the topography substrate used: black = 0 S; red = 50 S; blue = 150 S; green = 250 S; turquoise = 350 S; purple = 450 S.

To study cellular response towards wrinkles, osteoblasts were seeded on the surfaces and allowed to attach and spread for 2 days (Fig. 5A). The cell cytoskeleton and the formation of focal adhesion contacts were analyzed using immuno-labeling for fluorescent staining and confocal laser scanning microscopy (CLSM). Large differences were observed between the structured surfaces and the non-wrinkled control. On the control ^A0^C_W0_ (Amplitude 0 nm; Wavelength 0 nm) and ^A13^50S_W215_ substrates (50 s plasma oxidation; Amplitude 13 nm; Wavelength 215 nm), osteoblasts had more well defined dash-like vinculin spots (typical for mature focal adhesions) compared to the dot-like (transient) vinculin spots found for osteoblasts cultured on the other structured surfaces. Focal adhesions for cells plated on the structured substrates: ^A133^150S_W263_, ^A243^250S_W806_, ^A299^350S_W1008_ and ^A349^450S_W1239_ became less numerous, elongated and oriented themselves along the major cell axis, co-aligned with stress fibers (Fig. 5A). Although the wrinkles with larger features were not stimulating initial cell adhesion/spreading, the cell morphology shows that osteoblasts were better aligned and more elongated on the structured surfaces as compared to cells on the ^A0^C_W0_ and ^A13^50S_W215_ surface displaying the “flat” and smaller features, respectively which is in agreement with the gradient approach. More pronounced aligned features affect cell responses by regulating focal adhesion and stress fiber orientation, which define cytoskeleton organization and cell shape. These outcomes were similar to those reported in previous studies, where surface patterning induced cell alignment and elongation^30^. The osteoblasts adhered on ^A13^50S_W215_ were not prone to be influenced by the surface features, most likely the substratum is perceived as “flat” due to the small features.

For a better understanding of cell behavior on different substrates, focal adhesion area per cell (FA area/cell; μm^2^/cell) was determined by a quantitative analysis of the positively stained focal adhesions. Likewise, focal adhesion orientation and cell alignment were determined measuring the angle of focal adhesion and cell relative to the direction of the wrinkles using ImageJ software (Fig. 5B–D). Less FA area/cell was present with increasing amplitude, wavelength and aspect ratio (Fig. 5B). For the amplitude and wavelength the FA area/cell decreased until reaching a minimum while for the aspect ratio, as shown in Fig. 4B, a linear trend was observed (R^2^ = 0.992; Fig. S5). These observations depict a different situation as was shown for the gradients. With the uniform wrinkles, a fixed possible number of wavelength-amplitude combinations are obtained and these do not feature the broader range as obtained for the gradients. Figure 5C shows that FA orientation first increases and then decreases with increasing amplitude, wavelength or aspect ratio. It shows that more pronounced surface features have a positive effect on the alignment which is counteracting the focal adhesion behavior. It was found that the percentage of aligned cells has a comparable trend with FA orientation (Fig. 5D) indicating that aligned nanotopographies direct the cell alignment by influencing the direction of the focal adhesions which improves the osteoblast alignment. The process of alignment here occurs via a much more subtle process than the more physical approach of microstructures which physically entrap cells in a confined space and thereby introduce an orientation effect^39^,^40^. Comparison of focal adhesion characteristics showed a positive correlation of focal adhesion orientation with cell alignment. Additionally, a negative correlation between cell elongation and focal adhesion area per cell (Fig. 5C) was observed. This also agrees with cellular sequences of events as cell elongation and alignment are preceded by focal adhesion alignment^35^,^41^. The cell responses on the gradients displayed similar behavior.

**Figure 6:**
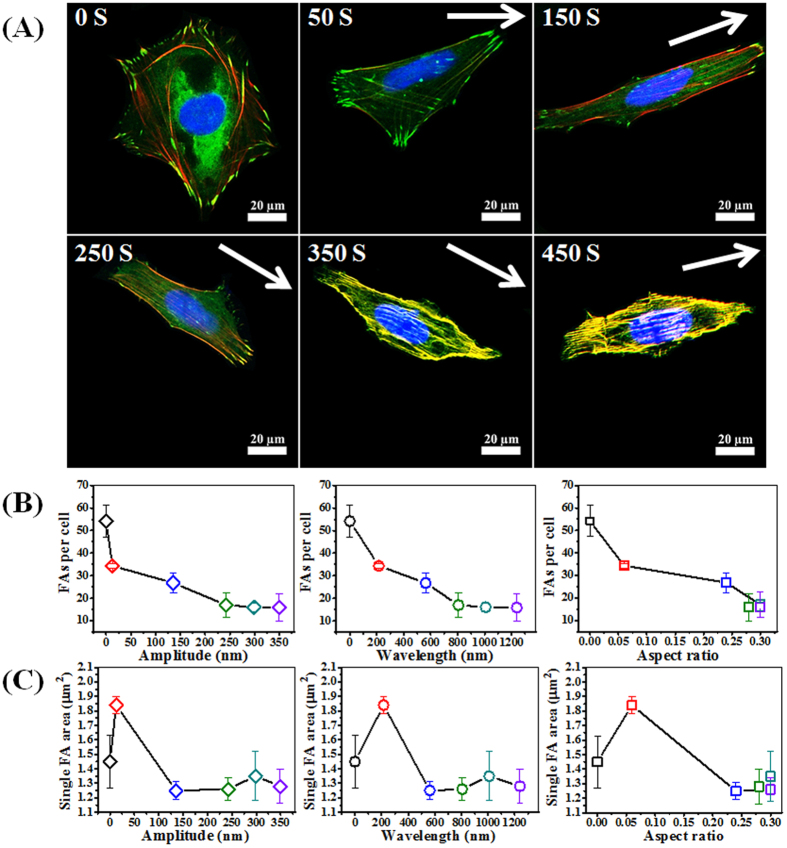
(**A**) Fluorescent staining of single osteoblast for different wrinkles prepared by varied plasma oxidation times (green staining is vinculin; red staining is F-actin, visualized by TRITC-phalloidin staining and blue staining is nucleus, stained by DAPI). (**B**,**C**) Dependence of focal adhesions per cell (**B**) and single focal adhesion area (**C**) on amplitude, wavelength and aspect ratio, respectively. Data are reported as mean ± standard deviation (SD) (n = 100 ~ 150 cells). Color coding corresponds to the topography substrate used: black = 0 S; red = 50 S; blue = 150 S; green = 250 S; turquoise = 350 S; purple = 450 S.

From the single cell images on the uniform wrinkles (Fig. 6A), cell shape and focal adhesion can be observed more clearly. It was found that cell and nuclei elongated with increasing wrinkle features. The cytoskeleton of osteoblasts on ^A0^C_W0_ and ^A13^50S_W215_, ^A135^150S_W263_, and ^A243^250S_W806_ was well spread and contained well-defined actin stress fibers, while the cytoskeleton on ^A299^350S_W1008_ and ^A349^450S_W1239_ appeared less organized and more diffuse. A dense network of F-actin was observed for the osteoblasts cultured on the ^H0^C_W0_ and ^H13^50S_W215_ surfaces, while parallel stress fibers of lower density were observed for the elongated cells on the other structured surfaces. It suggests that nanotopography exerts a significant influence on the organization of the F-actin cytoskeleton. The FAs and their distribution, visualized by immunofluorescence staining of vinculin, could be observed clearly for cells cultured on ^A0^C_W0_ and ^A13^50S_W215_, ^A135^150S_W263_, and ^A243^250S_W806_ samples. In contrast, a higher density of larger and uniform FAs was observed on the ^A0^C_W0_ and ^A13^50S_W215_ surfaces, whereas cells plated on the other structured substrates form numerous small and oriented focal adhesions. From Fig. 6B, it was found that focal adhesions per cell decreased with increasing the amplitude, wavelength or aspect ratio. However, from Fig. 6C, it was found that single focal adhesions area first increased and subsequently decreased with increasing amplitude, wavelength or aspect ratio. Therefore, compared to Fig. 5B, the reduction of focal adhesion area per cell is mainly due to the reduction of the number of focal adhesions per cell.

**Figure 7:**
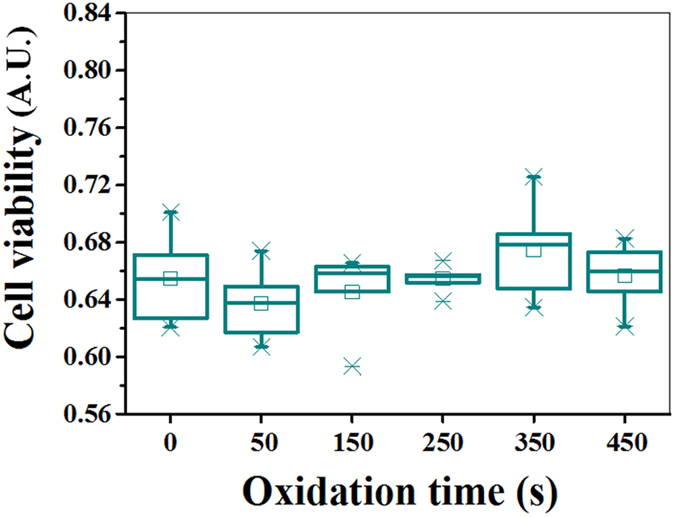
Cell viability of the osteoblasts cultured on the wrinkle PDMS substrates with different oxidation times using an XTT assay and analyzing the difference in absorbance (A.U.). 0 S (flat surface) is considered as the reference to which the structured nanowrinkles of different dimensions are compared to. Data are mean ± SD (n = 5).

To examine the cell viability, the osteoblasts were cultured onto the wrinkle PDMS substrates with different oxidation time for 2 days and analyzed using an XTT assay. From Fig. 7, it was found that cell viability on different nanostructured samples had no significant difference as compared to the flat control (0 S). This suggests that although there are more rounded cells and cells with less organized and more diffuse cytoskeleton on wrinkle substrates, this does not affect the cell viability.

## Conclusions

We developed a novel approach where well-defined surface topography gradients allowed us to determine relative feature contribution towards cell adhesion and alignment more efficiently and accurately as shown with osteoblasts as an initial test case. The main trends that **1)** cell alignment efficiency increasing at the expense of cell adhesion and **2)** a specific combination of features is responsible influencing the cell behavior are similar for both the uniform wrinkle substrates and the gradients. However, the gradients display that the linear trend of focal adhesion with aspect ratio is incorrect which only became apparent as the gradient substrates provide more wavelength/amplitude combinations for similar aspect ratios while the uniform substrates do not. Additionally, the number of cell culture experiments for the gradients (4) was less than for the uniform wrinkles (6) and provided more data which gave rise to better assessment of cell behavior at nanostructured interfaces. The osteoblast culture experiments show that the osteoblast behavior is influenced by small changes in surface topography. Less focal adhesion area per cell with increasing wrinkle features (i.e. amplitude, wavelength), while focal adhesion orientation and cell alignment initially increased with increasing wrinkle features and subsequently decreased again. It was identified that wrinkles with 130–180 nm amplitude and 550–730 nm wavelength are the most favorable topography for improving the osteoblast alignment while amplitudes of 15–45 nm and wavelengths of 400–520 nm are required to have an optimal combined parameter response to direct osteoblast behavior. Using the model system as developed here, prospective application of this idea will enable rapid acquisition of data for cell-biointerface interactions in order to understand the relationship between cell behavior and surface properties, required for the development of more effective biomaterials and applications in tissue engineering.

## Methods

### PDMS preparation

PDMS elastomer samples were obtained by mixing the prepolymer and cross-linker (Sylgard 184, Dow Corning) at a 10:1 ratio by mass. The 33 g mixture was vigorously stirred with a spatula, placed under vacuum for 15 min to degas, and deposited on a carefully cleaned, plain petri dish with a thickness of ~3.0 mm, before curing at 70 °C in a vacuum oven for overnight to crosslink into an elastomer.

### Preparation of uniform wrinkle and gradient thereof

The PDMS slab obtained was cut into strips of 3.0 × 1.5 cm. They were stretched uniaxial in a customer-made apparatus to a strain of 30% of their original length. For uniform wrinkle, after the stretched substrates were oxidized between 50 and 450 s in air plasma at 0.2 mbar (Plasma Activate Flecto 10 USB, maximum intensity), the stress was removed which induces wrinkle formation. For wrinkle gradient, stretched PDMS is partly covered with a mask (angle: 30°, length: 1 cm). The approach was based on previous investigation by Hiltl *et al.*^12^ only here uni-directional gradients were developed rather than multi-directional surface features. Using the same conditions with uniform wrinkle, the stress was removed which induces wrinkle gradient formation with different wavelength and amplitude. All samples were post-treated with plasma for 10 minutes to ensure that the surface is fully oxidized and that surface chemistry as well as stiffness is equal for all samples.

### AFM characterization

AFM images were obtained using a commercial atomic force microscope (Nanoscope V Dimension 3100 microscope, Veeco, USA) operating with the tapping mode in air.

### Cell adhesion studies

SaOs (human osteosarcoma cell line expressing wild type p53 and Rb, but lacking p16) were used for the cell adhesion studies. The growth medium consisted of Dulbecco’s modified Eagle medium containing 1000 mg/L glucose, GlutaMAX, 10% (v/v) fetal bovine serum and ascorbic acid 2-phosphate. The cells were incubated at 37 °C and 5% CO_2_.

All circular PDMS samples (Ø14 mm) were treated with 70% ethanol for sterilization and placed in 24-wells microtiter plates for 2 hour, after which the SaOs cells were seeded onto the samples in 24-well plates at a density of 4 × 10^4^ cells/well for cell adhesion. All plates were stored in an incubator at 37 °C and 5% CO_2_ for two days.

To observe cell adhesion, the SaOs were fixed with 3.7% paraformaldehyde solution in PBS for 15 minutes followed by washing for two times in PBS, permeabilized with 0.5% Triton X-100 in PBS for 3 minutes and blocked with 5% fatty-acid free BSA in PBS for 30 min to avoid non-specific binding. The primary antibodies against vinculin (mouse) and fibronectin (rabbit) used for immuno-staining were diluted in 1% BSA in PBS and the specimens were incubated for 1 hour. After the incubation period the specimens were washed 3 times for 5 minutes in 1% BSA in PBS. The second antibodies were added together with DAPI and TRITC-phalloidin and allowed to incubate for 1 hour at room temperature. The samples were washed twice in 1% BSA in PBS and once in PBS, all for 5 minutes. Cells were observed using a LEICA TCS SP2 CLSM equipped with 40 × NA 0.80 and 63 × NA0.90 water immersion objectives.

Image analysis of vinculin was done by Focal Adhesion Analysis Server^42^ and ImageJ software to measure average area per cell, sites per cell, average area per focal adhesion and focal adhesion orientation. Image analysis of aligned cell was done by ImageJ software. Cells were considered aligned if the angle between the long axis and the wrinkle was less than 10° 31. The Focal adhesion orientation is defined as cos θ, and θ is an angle of focal adhesion relative to the direction of the wrinkles. This index ranges from 1 (all focal adhesions are oriented in parallel) to 0 (focal adhesions are oriented randomly).

Cell viability was analyzed using an XTT assay (Applichem A8088). Briefly, on day 2, 200 μL of XTT reaction mixture (0.1 mL activation reagent and 5 mL XTT reagent for one plate) was added to each well and samples were incubated at 37 °C in a humidified atmosphere of 5% CO_2_ for 2 h. The 200 μL mixtures were added to a 96-well plate and the absorbance at 485 and 690 nm was recorded on the microplate reader. Experiments were performed in quintuplicate.

## Additional Information

**How to cite this article**: Zhou, Q. *et al.* Directional nanotopographic gradients: a high-throughput screening platform for cell contact guidance. *Sci. Rep.* **5**, 16240; doi: 10.1038/srep16240 (2015).

## Supplementary Material

Supplementary Information
