# Supplementary material for: Directional nanotopographic gradients: a high-throughput screening platform for cell contact guidance
Source: Sci Rep. 2015 Nov 17;5:16240. doi: 10.1038/srep16240 (PMC4647116; doi:10.1038/srep16240)
Supplement: Supplementary Information [file srep16240-s1.pdf]

SUPPORTING INFORMATION

Directional nanotopographic gradients: a high-throughput screening platform for cell contact guidance

Qihui Zhou,[a,b] Philipp T. Kühn, [a,b] Thirsa Huisman,[a] Elsje Nieboer,[a] Charlotte van Zwol,[a] Theo G. van Kooten,[a,b] and Patrick van Rijn[a,b,c]*

[a] Biomedical Engineering Department-FB40, University of Groningen, University Medical Center Groningen, A. Deusinglaan 1, 9713 AV, Groningen, The Netherlands.

[b] W.J. Kolff Institute for Biomedical Engineering and Materials Science-FB41, University of Groningen, University Medical Center Groningen, Antonius Deusinglaan 1, 9713AW Groningen, Netherlands.

[c] Zernike Institute for Advanced Materials, University of Groningen, Nijenborgh 4, 9747 AG, Groningen, Netherlands.

S1: Experimental setup and approach for surface wrinkle gradient formation

S2: Details on sub-nano wrinkle features

S3: Images for focal adhesion area/cell determination

S4: Analysis of surface features reached with longer oxidation times

S5: Linearity analysis of focal adhesion area/cell on non-gradient substrates

S1: Experimental setup and approach for surface wrinkle formation

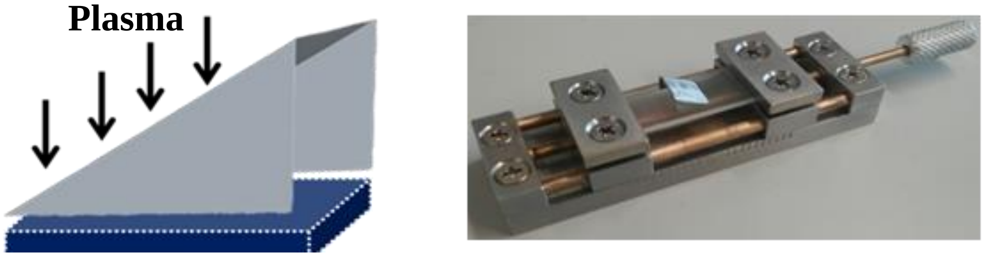

Figure S1. Schematic diagram and picture of formation of wrinkle gradient by air plasma oxidation.

S2: Details on sub-nano wrinkle features

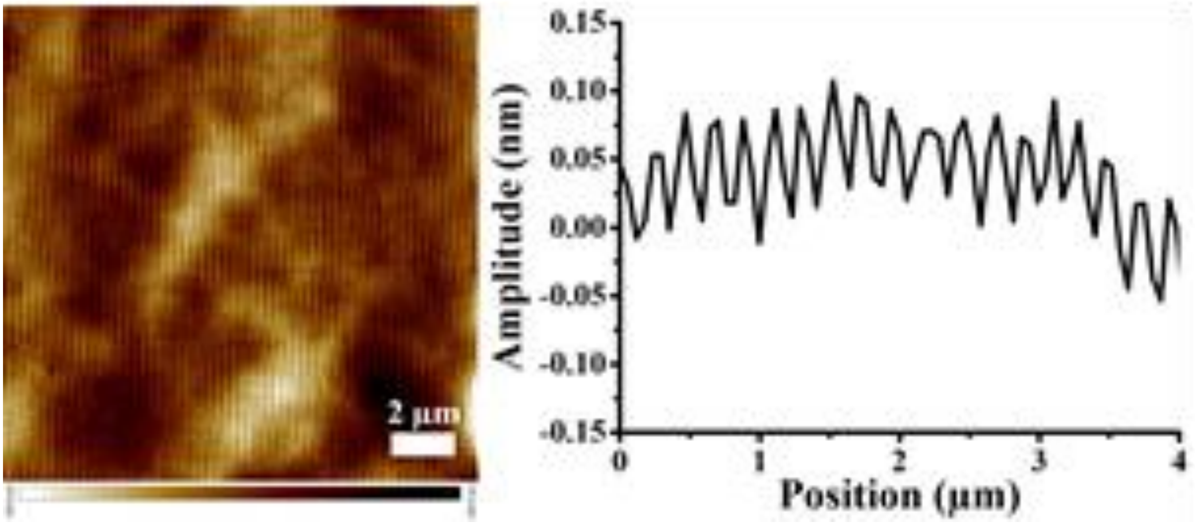

Figure S2. Zoom of surface features on position 0 cm on gradient formed using 50 s plasma treatment determined by atomic force microscopy.

S3: Images for focal adhesion area/cell determination

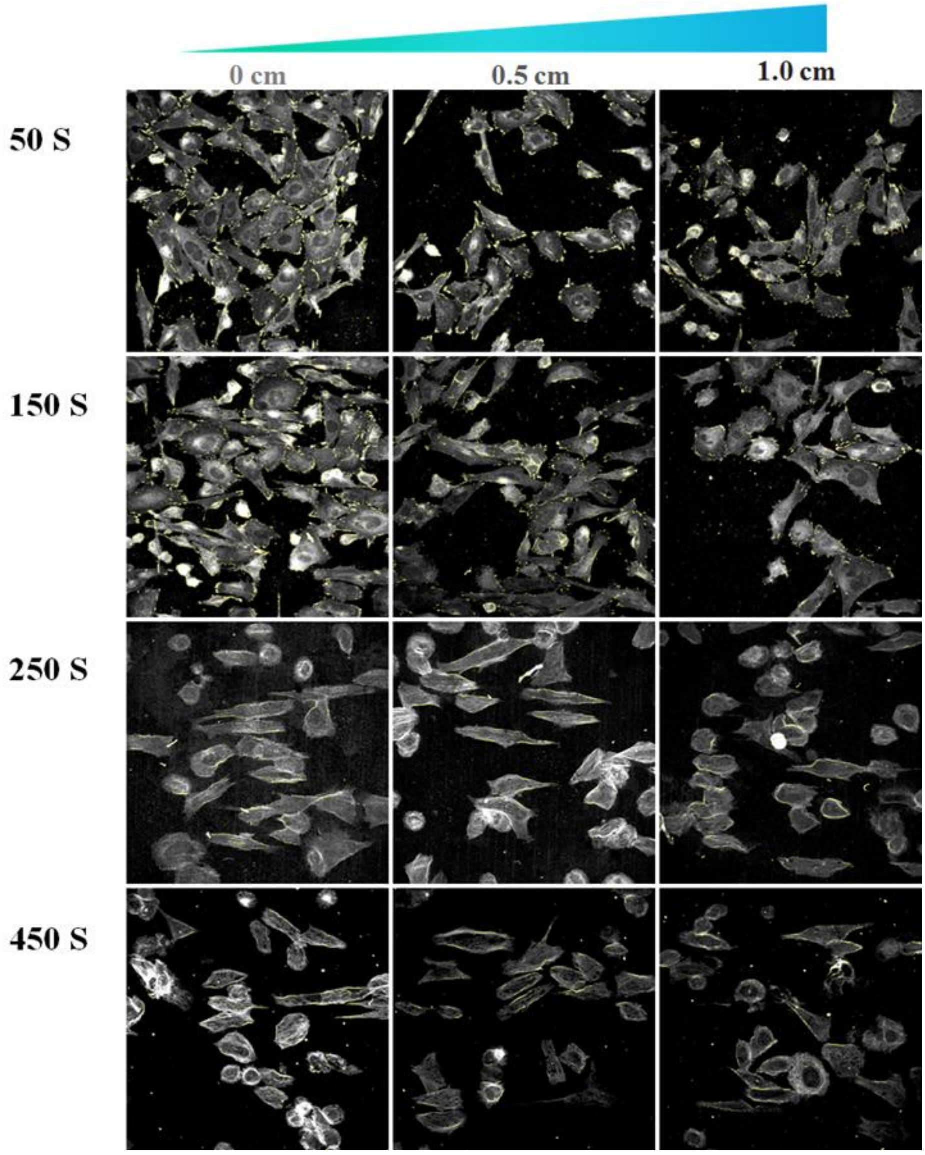

Figure S3. The highlight of focal adhesion contact points via Focal Adhesion Analysis Server (http://faas.bme.unc.edu/upload?static=1&max_adhesion_count=5000) for SaOs after 2 days cultivation on gradient surfaces.

S4: Analysis of surface features reached with longer oxidation times

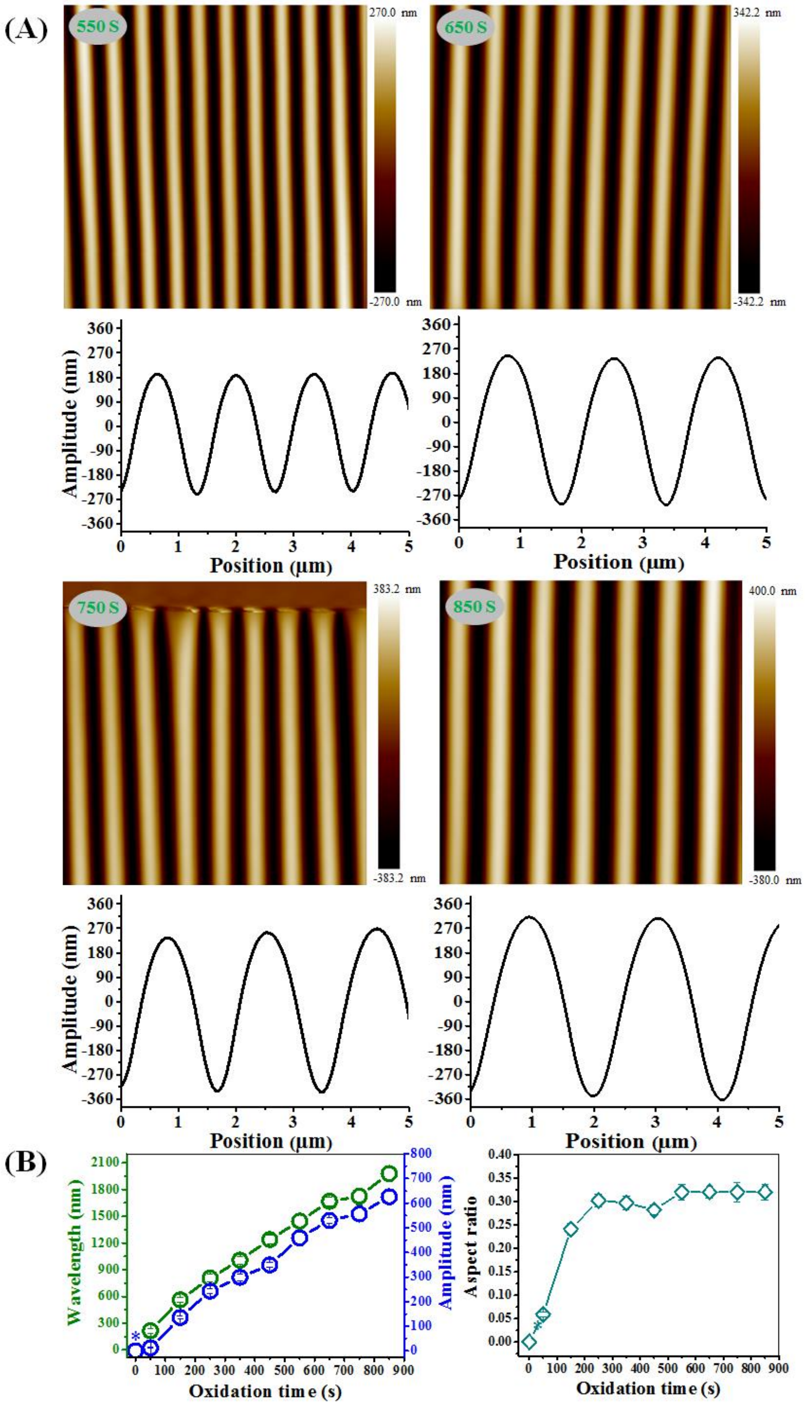

Figure S4. Dependence of the wavelength, height and aspect ratio of created wrinkles on the plasma oxidation time from 0 s-850 s with a fixed initial strain (30% substrate elongation). * indicates approximate value.

S5: Linearity analysis of focal adhesion area/cell on non-gradient substrates

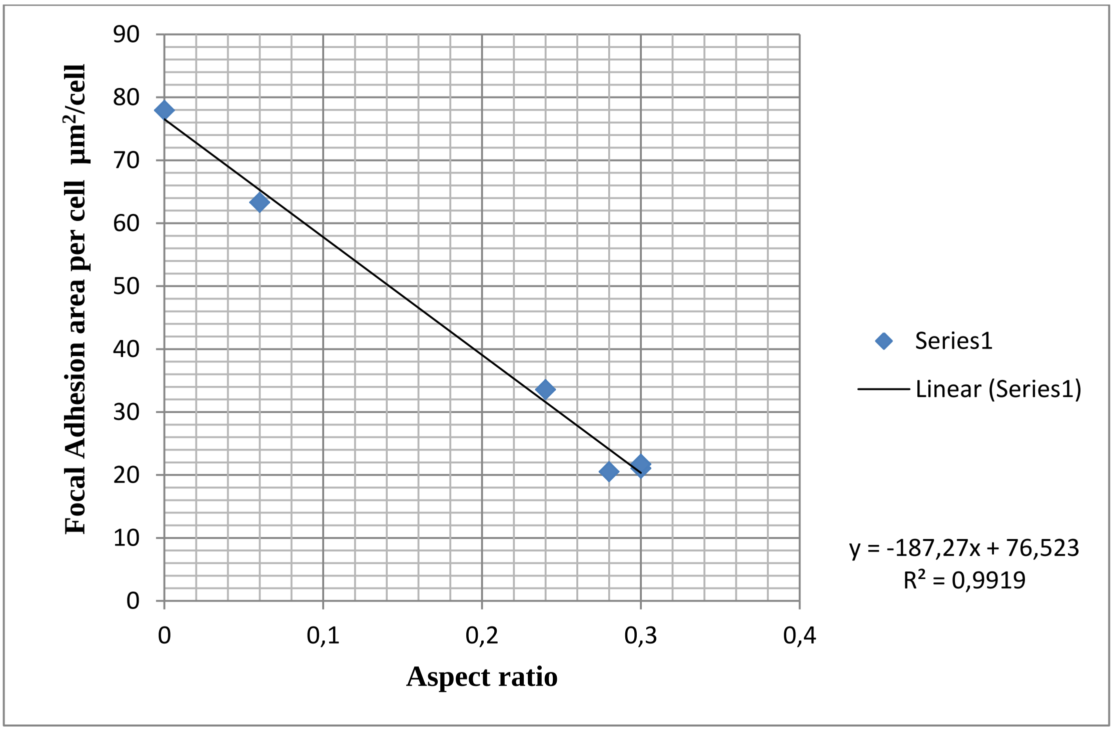

Figure S5. Determining the degree of linearity of wrinkle aspect ratio vs focal adhesion area/cell shows an R^2-value of 0.992 indicating that the trend can indeed be perceived as linear.
